# Probabilistic Load Forecasting for Building Energy Models

**DOI:** 10.3390/s20226525

**Published:** 2020-11-15

**Authors:** Eva Lucas Segarra, Germán Ramos Ruiz, Carlos Fernández Bandera

**Affiliations:** School of Architecture, University of Navarra, 31009 Pamplona, Spain; elucas@unav.es (E.L.S.); cfbandera@unav.es (C.F.B.)

**Keywords:** probabilistic load forecasting, white-box models, building energy models, weather forecast, uncertainty analysis, monitoring, reliability

## Abstract

In the current energy context of intelligent buildings and smart grids, the use of load forecasting to predict future building energy performance is becoming increasingly relevant. The prediction accuracy is directly influenced by input uncertainties such as the weather forecast, and its impact must be considered. Traditional load forecasting provides a single expected value for the predicted load and cannot properly incorporate the effect of these uncertainties. This research presents a methodology that calculates the probabilistic load forecast while accounting for the inherent uncertainty in forecast weather data. In the recent years, the probabilistic load forecasting approach has increased in importance in the literature but it is mostly focused on black-box models which do not allow performance evaluation of specific components of envelope, HVAC systems, etc. This research fills this gap using a white-box model, a building energy model (BEM) developed in EnergyPlus, to provide the probabilistic load forecast. Through a Gaussian kernel density estimation (KDE), the procedure converts the point load forecast provided by the BEM into a probabilistic load forecast based on historical data, which is provided by the building’s indoor and outdoor monitoring system. An hourly map of the uncertainty of the load forecast due to the weather forecast is generated with different prediction intervals. The map provides an overview of different prediction intervals for each hour, along with the probability that the load forecast error is less than a certain value. This map can then be applied to the forecast load that is provided by the BEM by applying the prediction intervals with their associated probabilities to its outputs. The methodology was implemented and evaluated in a real school building in Denmark. The results show that the percentage of the real values that are covered by the prediction intervals for the testing month is greater than the confidence level (80%), even when a small amount of data are used for the creation of the uncertainty map; therefore, the proposed method is appropriate for predicting the probabilistic expected error in load forecasting due to the use of weather forecast data.

## 1. Introduction

In the era of the Internet of Things (IoT), virtual and physical environments are being closely linked and widely used in various areas of the industry. Among these areas, building operation and management is receiving more attention due to the growing development of intelligent buildings and smart grids [1,2]. Smart buildings integrate connected objects through systems that monitor and control a great variety of variables, such as indoor temperature, weather data, airflow rates and CO${}_{2}$ concentration. Sensors are fundamental devices in this new generation of buildings because they connect the simulation model with the real world and they enable appropriate management and adequate decision making [3]. In this field, the energy efficiency of buildings is one of the most important research areas: buildings represent almost 40% of the world’s total energy consumption and thus hold great energy-saving potential [4]. Load forecasting is one of the key elements of this new intelligent building and smart grid environment, where network solutions are used to optimize energy sources.

Accurate load forecasts for buildings allow the optimum management of buildings’ energy systems and low-voltage networks in different contexts, such as energy management systems [5,6], energy storage system control [7], demand response (DR) and demand-side management (DSM) [8] and the integration of distributed energy resources [9].

The prediction of building energy is key for the optimization of its management, and it falls into three general categories in the literature [10]: black-box or data-driven models, white-box or physical models and gray-box or hybrid models. Black-box models are mathematical models constructed from historical data and lack explicit link between model inputs and physical building parameters. In recent years, several reviews articles have studied the growing use of this type of prediction models for building energy prediction [11,12,13]. Following the classification proposed in the most recent review from Sun et al. [13], data driven approaches can be divided into statistical, which derive correlations between the variable of study and influential parameters, such as linear regression (LR) or time series analysis (ARMA and ARIMA) [14,15]; and machine learning (ML) approaches, which are a more advanced statistical methods and use prediction algorithms. ML includes, among others, support vector machine (SVM) [16], ensemble methods [17], deep learning [5] and the increasing and most common in the recent literature [13,18], artificial neural networks (ANN) [19,20,21]. White-box or physical models are based on physical principles and predict loads with detailed heat and mass transfer equations using simulation software such as EnergyPlus and TRNSYS. These software packages calculate building energy prediction based on building construction details, heating, ventilation and air conditioning (HVAC) design information, operation schedules and climate information [22,23,24]. Finally, gray-box or hybrid models, which are a combination of data-driven and physics-based models, use simplified physical descriptions but also require parameter estimation based on measured data [25,26].

Among these building energy prediction models, black-box-based models lack an understanding of the underlying parameters of the energy prediction and its behavior so they are not transparent [18]. However, white-box models allow to monitor the modeling and analyze the process step by step interpreting the results for different scales (whole building, thermal zones, etc.) and link them with the physics and architectural parameters of the buildings. While black-box models, such as ANNs, are commonly used for small-scale modeling tasks or assuming that the zone temperature distribution is uniform [27], white-box models are able to characterize large multi-thermal zones buildings. On the other hand, white-box models are more flexible to changes in the buildings characteristics or operation since they do not require the re-training of the model, avoiding problems of input data quality [28,29]. Once the model is developed, it can be more easily used for other applications like retrofit analysis or fault detection and diagnostics [30], or conversely, it is easy to exploit a white-box model to provide load forecast previously developed for other purposes.

The common factor among building load forecasting techniques is that their accuracy depends not only on the accuracy of the model itself but also on the accuracy of the predicted external inputs [31]. Among these driving factors, weather parameter forecasting is a fundamental element because it has a great influence on the building’s actual energy consumption but has inherent uncertainty. The literature recognizes the significant influence of the weather forecast on a building’s energy performance, especially outdoor temperature [32]. However, the impact of the uncertainty due to forecast weather data on building load forecasting is not well represented in the literature [33,34,35], and few studies have directly investigated its effect [31,32,36,37,38].

On the other hand, traditionally, a load forecast is generated with a point or deterministic approach, which means that a single expected value for the predicted load is provided. The problem is that this point load forecast is not able to properly consider and quantify the effect of its inherent uncertainties. Therefore, it is necessary to develop a tool to quantitatively describe the uncertainties of load prediction and to assess the risk of relying on these forecasts. Uncertainties in load forecasting can be addressed through probabilistic load forecasting (PLF), an approach that can provide future predictions with the associated prescribed probabilities. This probabilistic approach is more adaptable to the current energy context, where the dependence of load forecasting on its inherent uncertainties complicates reliable and efficient energy management [39].

Probabilistic load forecasting is gradually increasing in importance in the literature, especially after the Global Energy Forecasting Competition 2014 [40], since it can provide more comprehensive information for the energy management decision-making process. Hong and Fan [39] provided a review of the state-of-the-art in probabilistic electric load forecasting where they stated that it can be implemented in practically the same cases in which single-valued load forecasts are applied. For example, it has been used for electricity consumption prediction in buildings [41,42,43], but also for distributed renewable energy production forecasts, such as photovoltaic power generation or wind speed forecasts [44,45]; applications related to electric vehicles [46]; and the quantification of the power reserve of a microgrid [47].

The literature includes several studies with different approaches that incorporate weather uncertainty into PLF development to forecast the building’s load. Xu et al. developed a probabilistic load forecasting model using an artificial neural network (ANN) and probabilistic temperature forecasts. Their results showed that the probabilistic normal load forecasts had satisfactory accuracies, and the load forecasts based on one-day-ahead probabilistic weather forecasts were the best [48]. Dahl et al. presented an autoregressive heat forecast model with weather prediction input and concluded that ensemble weather predictions could improve supply temperature control in district heating area substations [49]. Zhao et al. used the Monte Carlo method (MCM) to pre-process meteorological forecast data to improve the accuracy of load forecasts provided by a support vector machine (SVM) model, and the forecasting results became closer to the actual data [34]. Similarly, Fan et al. also employed the MCM to calibrate the input variables of their proposed SVM cooling load prediction model with the aim of reducing the influence of the uncertainty of the input variables (weather parameters, among others). With calibrated inputs, this approach produced a more accurate prediction, which was closer to the load prediction based on measured data [50]. Although probabilistic load forecasting studies are increasing in the literature, they are mainly based on black-box models which cannot clarify the link between inputs and the forecasted building loads.

This research aims to fill this gap by providing a probabilistic load forecasting methodology that considers the weather prediction uncertainty using white-box models (building energy models, BEMs) instead of black-box models. The proposed methodology converts the point load forecast provided by a BEM into a probabilistic load forecast using historical data based on indoor and outdoor building monitoring. An hourly map of the uncertainty of the load forecast due to the weather forecast is provided for a specific building and weather forecast source. After applying the uncertainty map on the BEM outputs, the hourly load forecast is obtained with the probabilistic error due to weather forecast data, providing a tool that can help the building’s energy managers and network operators to make more informed decisions.

The load forecast is provided by a BEM (physics-based model), which is fed weather files that are generated using a methodology that respects the thermal history of the building. Different time horizons, from 1 day to 6 days ahead, were employed in this study to assess their influence on the probabilistic load prediction. The methodology was applied to a real case study, a building located in Gedved (Denmark), which is equipped with indoor temperature sensors and an on-site weather station. Following the recommendations of Agüera-Pérez [35], this research used a real weather forecast from an external provider instead of synthetic data [51] and six weather parameters instead of using only temperature [52] or temperature and humidity [36].

The main contributions of this research are: (1) a probabilistic load forecasting approach is provided based on white-box models, instead of black-box models; (2) an hourly uncertainty map is provided as an easy tool to represent the expected hourly probabilistic load forecast error due to weather forecast for a specific building and weather prediction source; and (3) a dedicated script is developed to generate the daily weather files that feed the building energy model.

This paper is organized as follows: Section 2 shows the proposed PLF methodology, including the simulation and the probabilistic process performed on the data generated by the BEM. Section 3 focuses on the description of the case study for which the methodology was implemented, and Section 4 shows the results, including the evaluation of the methodology. Finally, Section 5 and Section 6 present the discussion and conclusions, respectively.

## 2. Methodology

**Figure 1:**
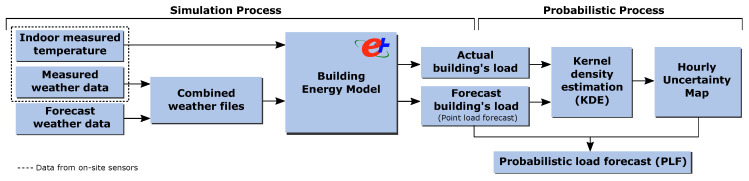
Components and steps of the proposed probabilistic load forecasting methodology based on white-box models (building energy model (BEM)).

This section presents the methods for the proposed probabilistic load forecast technique, which uses a BEM and accounts for weather forecast uncertainty. This technique requires two procedures, which are detailed in the following sections: the simulation process to determine the historical impact of the weather forecast data on the load provided by the BEM and the probabilistic processing of the simulation outputs. The overall approach of the proposed probabilistic load forecasting methodology is illustrated in the Figure 1.

Since the effect of the weather forecast on the energy model is considered in this methodology, all uncertainties related to the simulation must be minimized so that the results of the probabilistic analysis are valid. Section 2.1 discusses aspects such as the thermal history, the creation of weather files, the accuracy of the building energy model used, which accounts for the building’s internal loads and the preparation of outputs for the probabilistic analysis. Then, Section 2.2 details the probabilistic processing of the historical differences between the loads obtained from the observed and forecast weather data employed to obtain the uncertainty map, which is subsequently applied to the point forecast.

### 2.1. Simulation Process through a BEM

In the field of forecasting building loads, many studies have used statistical or machine learning approaches and reported low errors and good prediction accuracy. However, since these models were trained using historical data, their ability to adapt to changes in the building is limited. Physical models, which are based on physical and universal laws and equations, are more adaptive to changes [53]. These models facilitate an understanding of the thermodynamic aspects and interactions with the internal and external environments. The proposed methodology employs calibrated building energy models (BEMs) based on EnergyPlus simulation software [54,55] as the best representation of the behavior of the real building.

In order to compare the load differences using observed and forecast weather data, it is necessary to explain how the forecast and observed data are implemented in the BEM. In this regard, important roles are played by weather files, generated with both observed and forecast information; the indoor temperature, as the best representation of the internal loads of the building; and the simulation periods, which are very relevant when processing the thermal history in the simulation.

**Figure 2:**
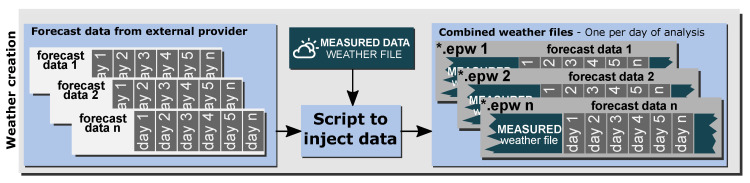
Weather file creation methodology.

To ensure the appropriate initial conditions in the simulation process, the weather files must be created with respect to the thermal history of the building for both the internal and external conditions. In this research, a procedure was developed to generate weather files in EPW (EnergyPlus weather file) format to meet this requirement. The Weather Converter [56] tool, provided as an auxiliary program by EnergyPlus, was used for the creation of all of these files by translating and extending typical weather data into the EPW format and making the necessary calculations for unavailable data. The source of the observed weather data is an on-site weather station installed in the building surroundings. Following the recommendations made by Agüera et al. in [35], real weather forecast data supplied by an external provider were used in this methodology, instead of using arbitrary forecasts based on synthetic data [57] or historical forecasts, which can be treated as perfect forecasts or modified by adding variations [36] that do not provide the real context of the building’s performance. The process of creating the weather file started with the collection of daily forecast weather data from the external provider. Then, one weather file was generated for each day with the measured weather information (historical data) and forecast data. The resulting file is called the combined weather file and contains both observed and forecast data. The process was implemented using a dedicated script that injects the forecast weather data into the historical weather data. Therefore, the combined daily weather files contain *n* days of forecast weather data, and the rest of the data correspond to the observed data provided by an on-site weather station. Figure 2 depicts the methodology.

**Figure 3:**
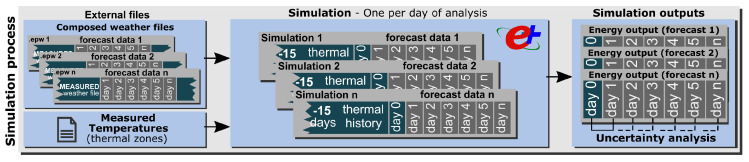
Simulation process methodology.

It is crucial that the BEM correctly reflects the past thermal behavior by taking into account all loads of the building (HVAC system, people, lighting, electric equipment, etc.). The best way to account for these loads is to use the building’s indoor temperature measurements via the sensors of the building management system (BMS). An external file with the actual indoor temperature is used by the simulation model as a dynamic set-point for the HVAC system. The simulation output is the energy demand required by the model to follow it. This is shown in Figure 3.

The probabilistic analysis was carried out by comparing the energy differences in the models when using the observed and forecast weather data. To obtain accurate energy differences, the use of the correct simulation period is very important. One simulation per day of analysis was executed since each day has its own weather file. For each day, the simulation was configured to run 15 days before the baseline day (day 0) with the measured weather data to capture the thermal history of the model. The loads on day 0 and *n* days of the forecast were obtained as the outputs of each simulation. These results (ordered and classified) were subsequently used in the probabilistic analysis.

The error of the point load prediction provided by the BEM when using weather forecast can be evaluated using three error metrics commonly employed in the forecasting literature: mean absolute error (MAE) (Equation (1)), which measures the average magnitude of the error in the units of the variable of interest; mean absolute percentage error (MAPE) (Equation (2)), which is a relative error measure that allows comparing the forecasts performance on different data sets; and the coefficient of determination ($R^{2}$) (Equation (3)), which allows to measure the linear relationship of the two patterns [58].

(1) $$MAE=\frac{1}{n}\sum_{i=1}^{n}\left|y_{i}-{\hat{y}}_{i}\right|,$$

(2) $$MAPE=\frac{1}{n}\sum_{i=1}^{n}\frac{|y_{i}-{\hat{y}}_{i}|}{{\hat{y}}_{i}}\times 100\%,$$

(3) $$R^{2}={\left(\frac{n\;\cdot \;\sum_{i=1}^{n}y_{i}\;\cdot \;\hat{y}-\sum_{i=1}^{n}y_{i}\;\cdot \;\sum_{i=1}^{n}\hat{y}}{\sqrt{\left(n\;\cdot \;\sum_{i=1}^{n}y_{i}^{2}-{\left(\sum_{i=1}^{n}y_{i}\right)}^{2}\right)\;\cdot \;\left(n\;\cdot \;\sum_{i=1}^{n}{\hat{y}}^{2}-{\left(\sum_{i=1}^{n}\hat{y}\right)}^{2}\right)}}\right)}^{2}$$

### 2.2. Probabilistic Load Forecast

The probability load forecast (PLF) proposed for this methodology was produced by applying the probability density function of residuals to the point forecast. This approach to producing the PLF was classified as output by Hong et al. in [39]. The point forecast provided by the BEM was converted to the PLF using historical data based on the observed and forecast weather data and the building’s indoor temperature.

First, the distribution of the residuals, which are the energy load differences provided by the BEM when it is fed the observed and forecast weather data, was studied through a probabilistic histogram. This is useful because it provides a straightforward visualization of the spread and the skewness of the data, the presence of outliers and the presence of multiple modes in the data. Second, to obtain a smooth curve that represents the data, a probability density estimation was performed. Many studies have used the normal distribution to estimate the density function, but it performs well only when the data follow a bell-shaped distribution. To avoid making assumptions about the distribution of the data, kernel density estimate (KDE) method was employed in this methodology, which is a non-parametric representation of the probability density function [45]. If sequence *X* consists of *N* 1-dimensional observations $x_{1},x_{2},\cdots ,x_{N}$, the KDE method estimates the actual probability density function *f* through the following function (4):

(4) $${\hat{f}}_{h}\left(x\right)=\frac{1}{n}\sum_{i=1}^{n}K\left(x,x_{i}\right)$$

where *K* is the kernel function, which is a non-negative symmetric function that integrated to one and has mean zero. There are many types of kernels but empirically is it not very relevant which one is employed [59]. In this case, Gaussian kernel is used to realize the KDE. It replaces each sample point with a Gaussian-shaped kernel and then obtains the resulting estimate for the density by adding these Gaussians. It can be expressed by (5) [45]:

(5) $$K\left(x_{1},x_{2},\sigma \right)=\frac{1}{\sqrt{2\pi }\sigma }e^{-\frac{{\left(x_{1}-x_{2}\right)}^{2}}{2\sigma^{2}}}$$

The σ>0 is the bandwidth, a smoothing parameter that influences the shape of the distribution. There are many bandwidth selection methods [60] and the advantages of using Gaussian kernel KDE is that it can calculate the bandwidth by a rule of thumb automatically [61].

**Figure 4:**
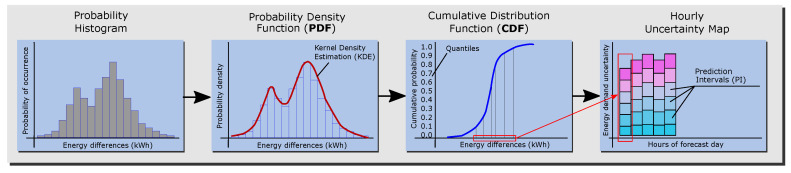
Process of the probabilistic load forecast.

The objective of this methodology is to obtain the expected probability that the load forecast error is below a certain value. The cumulative distribution function (CDF) or S-curve is an easily interpretable representation of the probability that the variable (here, the load forecast error due to the weather forecast) will be less than or equal to a certain value. Finally, from the CDF plot of each forecast hour, prediction intervals (PIs) of load forecast errors are extracted and transformed into an hourly map of uncertainty. The schema in Figure 4 shows the complete process.

The map of the uncertainty of the load forecast due to weather forecast data shows an overview of the probability that the load error is below a certain value for each hour. The map was constructed with the available hours ahead of the forecast time, and it is read as follows: for hour *n*, there is an x% probability that the energy demand error is less than *y* kWh. This map of uncertainty can then be applied to the load forecast provided by the BEM by applying the intervals of the energy demand error with their probabilities to the load forecast outputs of the model. In this way, the load forecast is obtained with the probability error due to weather forecast data, similar to a risk map. It can be used, for example, by the building’s energy manager to make a more informed choice according to the bearable risk.

Finally, for the evaluation of the methodology, two indicators commonly used in the related literature were employed for the prediction interval assessment: the prediction interval coverage probability (PICP) and the mean prediction interval width (MPIW) [62]. PICP measures the reliability of the predictions and shows the percentage of the real values that will be covered by the upper and lower bounds. The larger the PICP, the more likely that the real values are within the prediction interval. It can be defined as:

(6) $$PICP=\frac{1}{H}\sum_{i=1}^{H}C_{i},$$

in which *H* is the number of samples, and $C_{i}$ is a Boolean variable defined as follows:

(7) $$C_{i}=\left\{\begin{array}{cc} 1, & y_{i}\in \left[L_{i},U_{i}\right]\\ 0, & y_{i}\notin \left[L_{i},U_{i}\right], \end{array}\right.$$

where $L_{i}$ and $U_{i}$ are the lower and upper PI bounds of target $y_{i}$, respectively. PICP ranges between 0 and 100%. The prediction interval is considered valid if the PICP value is greater than the prediction interval nominal confidence (PINC=100(1−α)%), where α represents the probability of error.

High PICP values can be easily reached when the width of the prediction intervals (PIs) is large. However, large PIs have higher levels of uncertainty, and, thus, they are useless for decision making. Therefore, a complementary metric is required to assess the prediction interval widths: this metric is the Mean Prediction Interval Width (MPIW), which is defined as:

(8) $$MPIW=\frac{1}{H}\sum_{i=1}^{H}\left(U_{i}-L_{i}\right).$$

In conclusion, to make a suitable decision, small MPIW and high PICP values are desirable.

As mentioned in the methodology explanation, the validity of the results of the probabilistic analysis is closely related to the data selection and processing used to create the uncertainty map. The following section describes a case study in which the methodology was implemented, and it illustrates the importance of sensors for obtaining both the weather file and the indoor temperatures.

## 3. Description of the Case Study

**Figure 5:**
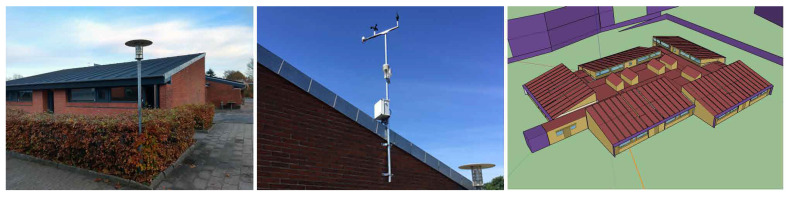
Library building from Gedved School, Denmark. Left: Outdoor image. Middle: Weather station installed on the building’s roof. Right: The building energy model (OpenStudio plugin for SketchUp [64]).

In this section, the case study that was used to apply the proposed methodology is presented. The test site is a public elementary school in Gedved, Denmark. This building is part of the EU-funded H2020 research and innovation project SmArt BI-directional multi eNergy gAteway (SABINA) [63]. Gedved School consists of 6 buildings and was built in 1979 and renewed in 2007. For this case study, the library was selected, which is a one-story building with a total surface area of 1138 m${}^{2}$. The main characteristics of the building are as follows: there are two brick layers with 150 mm insulation in between them for the facades; the windows are two-layer double-glazed with cold frames; the ceiling is insulated with 200–250 mm mineral wool for sloping and flat ceilings, respectively; and the floor is made up of concrete and contains 150 mm insulation underneath. Regarding HVAC systems, only heating is provided to the building, and it is connected for 24 h every day from October to May. Figure 5 shows an outdoor photograph of the library.

The building energy model used in this study was provided by the SABINA project and was developed using the EnergyPlus engine. In the load forecasting field, when using BEMs, it is necessary to take into account three main sources of uncertainty: BEM accuracy, building use and external conditions. In order to minimize the first uncertainty, this case study employed a calibrated BEM, obtained using a calibration methodology explained in the authors’ previous papers [58,65,66,67,68]. Regarding the building’s use, no uncertainty was consider in the indoor conditions since the model used indoor temperatures measured in each thermal zone by the BMS.

For the creation of weather files, the external conditions are required for both the observed and the forecast weather data. The observed weather data were obtained from a weather station installed on the building’s roof, which provides measurements with hourly intervals for atmospheric pressure, temperature, humidity, direct and diffuse irradiation, wind speed, wind direction and rainfall. Figure 5 shows the weather station location. The forecast weather information used in this study is a real forecast supplied by the commercial service Meteoblue [69]. This company uses a multimodel/machine learning approach to calculate the forecast weather data using both Meteoblue weather models (Nonhydrostatic Meso-Scale Modeling) and third-party models for the simulations. More information about Meteoblue’s forecast weather data process is available on its web page [69]. For this study, the weather forecast data were gathered at 09:00 on each day. For the time horizon, 6 days ahead of the weather forecast data were available for the present study.

The period in which all of the required data were available is from December 2018 to April 2020. The summer months (from June 2019 to September 2019) were not useful for the present study since no cooling system is installed in the building, and, therefore, only heating load forecasting was considered. Thus, the final period of the study is composed of 13 months, from December 2018 to May 2019 and from October 2019 to April 2020, so two complete winter campaigns (2018–2019 and 2019–2020) were analyzed.

First, the methodology is illustrated using a map of uncertainty generated from all 13 months of data to show how this probabilistic load forecast (PLF) method is implemented using a BEM. Then, the application of the uncertainty map to the heating load forecast provided by the BEM is presented for a random day. Finally, it is evaluated the ability of the proposed PLF methodology to predict the expected error in the heating load using the 12 first months of the period of the study to generate the map of uncertainty and the last month (April 2020) to test the method.

## 4. Results

In this section, the results of applying the proposed probabilistic load forecasting (PLF) methodology to a real test site are presented. As mentioned before, the methodology converts the point load forecast provided by a BEM, which is a single-value, into a probabilistic load forecast. Table 1 presents the quantitative errors of the point load forecast provided by the BEM for the different time horizons (from 1 to 6 day-ahead). It shows the error metrics MAE, MAPE and $R^{2}$ between the forecast and real heating load when using the forecast and actual weather data, respectively. The results showed, as expected, the increase in the error as the day ahead grows (MAE and MAPE values increased and $R^{2}$ decreased).

**Figure 6:**
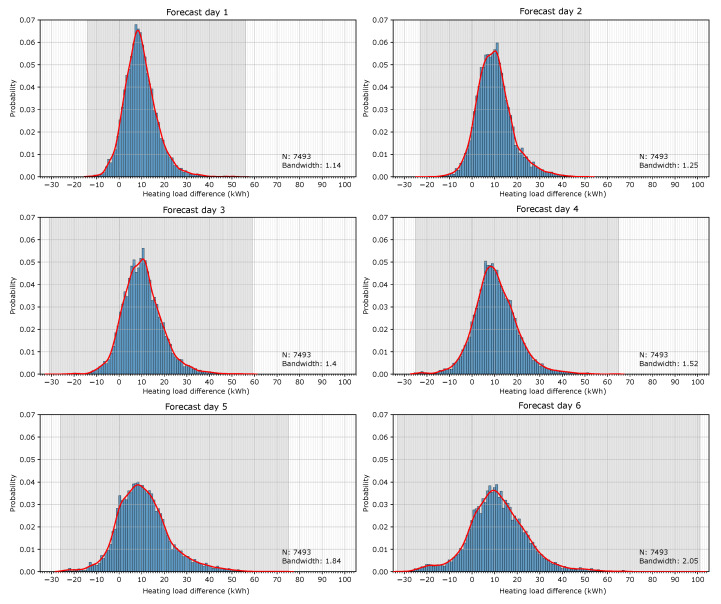
Probability histogram of the 6 forecast days and the Gaussian kernel density estimation (red line).

In order to show how the methodology is implemented, the whole period of study (13 months of 2 winter campaigns) was employed for the construction of the map of uncertainty. The differences in the hourly heating load between simulations with observed and forecast weather data were transformed into a probability histogram. This process was first performed for each full day-ahead in order to show the influence of the forecast time horizon on the heating load provided by the BEM. Figure 6 shows the histograms for the 6 forecast days. The highlighted gray area shows the spread of the variables for each forecast day. The graphs show that this gray area grows, which means that there are larger errors, as the forecast days increase. The errors in all the forecast days do not follow a symmetrical distribution with respect to the zero, and they all tend to skew toward the right, which means that the heating energy demand simulated with the forecast weather data is mainly overestimated. The graphs also present the probability density functions (PDFs) based on the Gaussian kernel density estimation (KDE) (red line).

**Figure 7:**
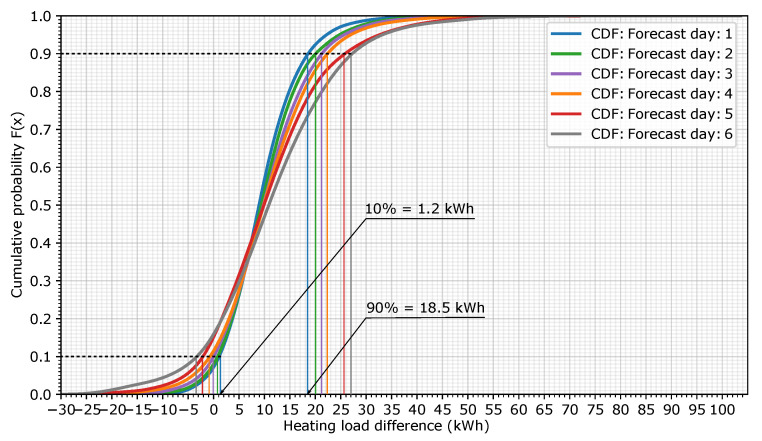
Cumulative distribution function (CDF) for the 6 forecast days.

The information in Figure 6 was transformed into a cumulative density function (CDF), which depicts the probability that an error in the heating load (in kWh) is below a particular value. Figure 7 shows the representation of the CDF for the six forecast days. For example, in this case, forecast day 1 has a 90% probability that the hourly load forecast error is below 18.5 kWh or a 10% probability that it is below 1.2 kWh (blue lines). The plot also shows that the heating load error increases as the forecast days increase. In this example, for the same 90% probability, the heating load error for forecast day 6 grows to 27 kWh.

The previous plots graphically show the hourly heating load error probability as a function of the full forecast day. In many applications of load forecasting, such as demand response or model predictive control, an hour-by-hour load error approach is more useful since most applications are implemented on an hourly basis. Thus, the same procedure was performed but based on an hourly approach from hour 1 to hour 144 with respect to the forecast time (09:00 h). The probability histogram, the fitted PDFs (KDE), and the CDF were calculated for all of the data that correspond to the first forecast hour, the second forecast hour and so on, with respect to the forecast time.

With the hourly CDF, the heating load error probability can be extracted as prediction intervals (in this case, intervals of 10%), making the probabilistic load forecast information more useful and applicable. For this purpose, a map that summarizes all of the hourly information was computed in order to present an overview of the heating load error due to the weather forecast as a function of the hours ahead of the forecast time. This generates the hourly uncertainty map of the heating load forecast due to weather data uncertainty for this specific building and weather forecast provider.

**Figure 8:**
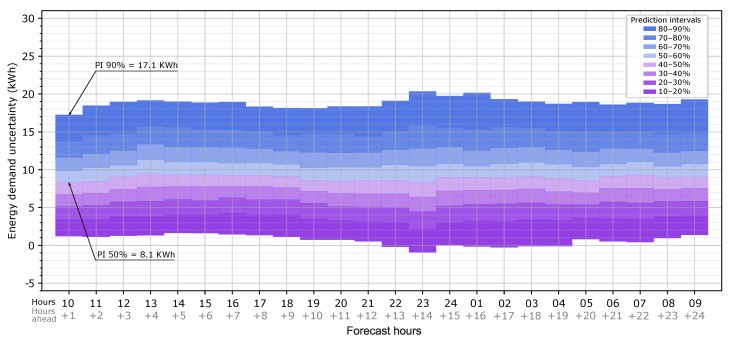
Probabilistic heating load forecast results: Hourly uncertainty map of the heating load forecast due to weather forecast data for the first 24 h ahead of the forecast hour (09:00 h).

The bound margins are obtained by a normal inverse cumulative distribution function for a desired probability index *x*, which, in this case, ranges from 10% to 90%. Figure 8 shows the uncertainty map for the first 24 h ahead of the forecast hour (09:00 h). For each hour, the expected heating load error due to weather forecast uncertainty can be extracted. For example, at 10:00 on forecast day 1, there is a 50% probability that the error in the heating load is less than 8.1 kWh, or, being more conservative, there is a 90% probability that the error is less than 17.1 kWh. In other words, the use of the forecast weather data leads to an overestimation of the heat load of 8.1 kWh and 17.1 kWh, respectively. The map shows that the heating load predicted with the forecast weather is likely to be overestimated with respect to the observed weather data for all of the forecast hours since the prediction intervals are mainly above 0. When the map is applied to the forecast heat load provided by the BEM, this overestimation must be considered inverted: there is a 50% probability that the simulation with forecast weather data provides 8.1 kWh in excess.

**Figure 9:**
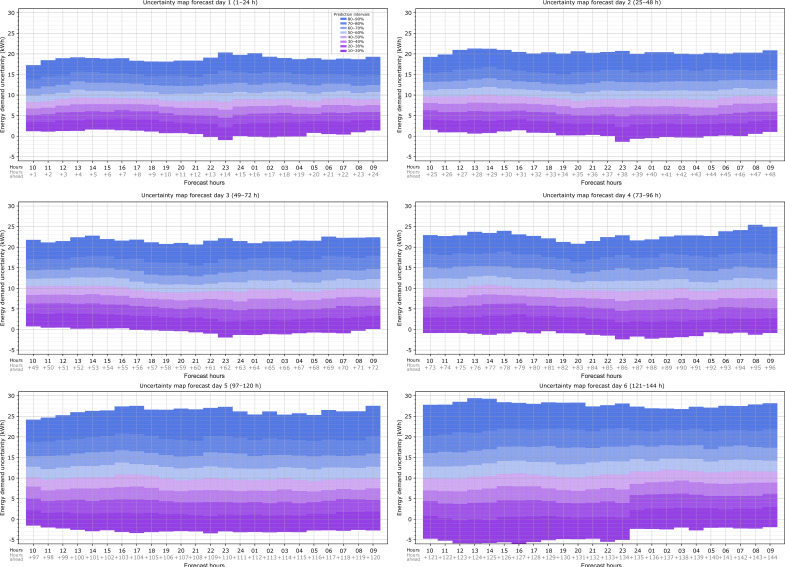
Probabilistic heating load forecast results: Hourly uncertainty map of the heating load forecast due to weather forecast data for 1 to 6 days ahead.

This map can be computed for as many forecast hours as there are available. Figure 9 shows the uncertainty map for the 144 h forecast hours available in this case study, divided into the six forecast days. As expected, the load uncertainty increases as the forecast hours increase, and this is reflected in these maps: for example, at 10:00 on forecast day 6 (121 h ahead of the forecast time), there is a 50% probability that the heat load error is lower than 10 kWh, which is 1.9 kWh higher than that for the same probability at 10:00 on forecast day 1 (1 h ahead of the forecast time), and there is a 90% probability that the heat load error is lower than 27.9 kWh, which is 10.8 kWh higher than that for the same probability at 10:00 on forecast day 1. The graphs also show that the width of the prediction intervals in the results grows as the forecast hours increase since, as seen in the histograms for the daily analysis (see Figure 6), as the forecast days increase, the dispersion of the results also increases.

**Figure 10:**
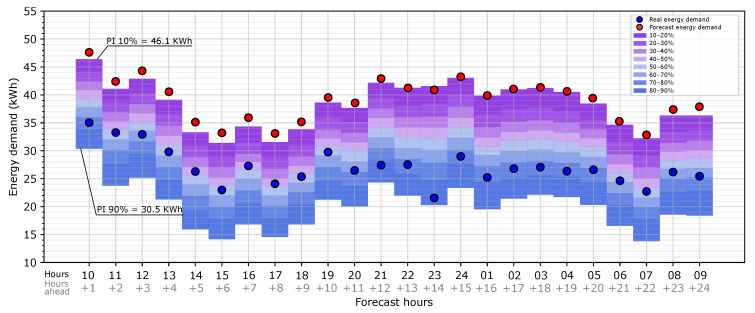
Application of the map of the heating load forecast uncertainty due to forecast weather data using the whole period of the study for a random day: 2 January 2020.

Once the map of uncertainty is obtained, it can be applied to the heating point load forecast provided by the BEM. To illustrate this, the previous map is shown for a random day (2 January 2020). Figure 10 shows the heating load forecast with the uncertainty map for prediction intervals from 10% to 90% implemented for 2 January 2020 and for 1 day ahead (from 1 to 24 h ahead). In the graph, the red dots represent the load forecast provided by the BEM for this specific day. The load uncertainty is represented as prediction intervals around the heating load forecast. When the predictions intervals (PIs) are below the heating load forecast, the energy demand is overestimated, and if they are above, it is underestimated. For the case study, the graph shows that the prediction intervals tend to be below the forecast energy demand, which means that it is much more likely that the model overestimates the heating load due to the weather forecast. In order to clarify how the graph is read, we provide the following example. At 10:00, which is 1 h ahead of the forecast time, the forecast heating load provided by the BEM is 47.6 kWh (red dot), and there is a 90% probability that the real heating load (with the observed weather data) is more than 30.5 kWh; as another way to analyze it, there is an 80% probability (prediction intervals between 10% and 90%) that the real heating load is higher than 30.5 kWh and lower than 46.1 kWh. According to the risk that the energy manager can accept, higher or lower probability intervals can be used for decision making. In the graph, the blue dots represent the real heating load, which is the result of the simulation with the observed weather data. For this example, all the dots are within the PI, which means that, for this case, this procedure provided a good prediction of the error in the heating load forecast due to the weather forecast data.

**Figure 11:**
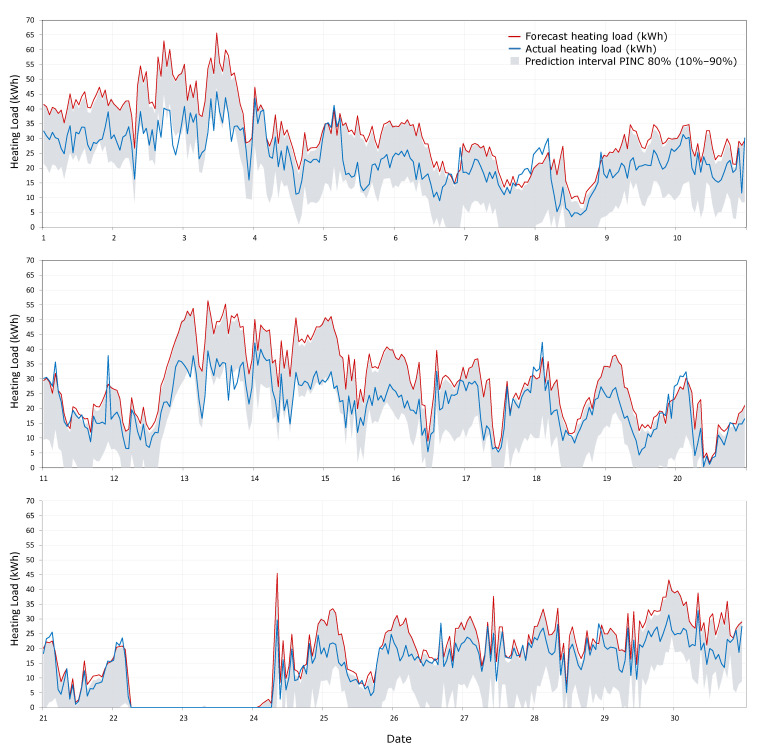
Results for the validation of the probabilistic load forecasting methodology for the testing period (April 2020). Above: from 1 to 10 April 2020; middle: from 11 to 20 April 2020; and below: from 21 to 30 April 2020.

The evaluation of the ability of the methodology to predict the load forecast error due to the weather forecast was performed for the 1 day-ahead data since it is the most commonly used time horizon in load forecasting. PICP and MPIW indicators were used to perform the assessment, and prediction intervals from 10% to 90% were considered. The validation of the methodology was performed using the first 12 months of the data (from December 2018 to May 2019 and from October 2019 to March 2020) to generate the uncertainty map and the most recent month, April 2020, to test the prediction interval accuracy. This month has a range of actual hourly heating loads from 0 on the mild days to 45.8 kWh on the coolest days. The PICP result is 83.1%, which is greater than the confidence level (PINC = 80%), and the MPIW is 17.5 kWh. Figure 11 shows the graphs of the results of the testing period (April 2020). In order to facilitate its interpretation, the graph for the whole month is divided into three parts. The actual loads are indicated by the blue line, the heating load forecast is the red line and the gray area represents the 80% prediction interval (from 10% to 90%). It can be seen that the prediction intervals generated with the proposed method cover the actual value most of the time.

Previous evaluation is performed using the 12 previous months prior to the testing month (April 2020) to generate the uncertainty map, but, how does the amount of data affect the uncertainty map and the results? In order to analyze this influence an analysis is performed gradually increasing the amount of data used for the creation of the uncertainty map. Maintaining April 2020 as the testing period, the uncertainty map is created using only the previous month (March 2020), the previous two (February 2020–March 2020) and so on. Table 2 presents the results. It shows that in all the cases the PICP values are higher than the confidence level (80%). It is remarkable that PICP values increase as months closer to the testing period are used for the creation of the uncertainty map, despite having a lesser amount of data, but MPIW values increase as well providing higher prediction intervals widths. Therefore, the results are robust and similar despite the number of months employed to build the uncertainty map.

## 5. Discussion

In the current energy context, load forecasting is becoming increasingly widespread, and the influence of the uncertainties that affect the prediction of future energy demand must be taken into consideration. One of the most important uncertainties is the weather forecast, but traditional point load forecast cannot properly reflect the effect of this uncertainty. This paper aims to provide a methodology that calculates the probabilistic load forecast while accounting for the inherent uncertainty in forecast weather data. Recently, PLF approaches have gained importance in the literature but focused mainly in black-box models. In order to fill this gap, this research provides a PLF methodology based on white-box models (BEMs). This methodology generates an hourly map of the uncertainty of the load forecast, which allows the point load forecasting provided by the BEM to be converted into a probabilistic load forecast. The map of uncertainty is created through a probabilistic analysis of the impact that the weather forecast has on the building’s load, which is provided by the BEM using data from the past. This analysis is possible thanks to the use of an accurately calibrated BEM and data gathered from different sources: the indoor conditions are provided by the sensors of the building’s monitoring; the observed outdoor conditions are supplied by an on-site weather station; and the weather forecast is provided by an external service.

A case study in a real school building in Gedved, Denmark, is presented. Thirteen months of data from the building’s monitoring, weather station and weather forecast were available, which corresponds to two complete winter campaigns. As a result that this building lacks a cooling system, the study was performed for the heating load forecast, and only the months in which the systems were actuated were considered.

Unlike other studies from the literature, this research employed a physics-based model, which is a calibrated BEM developed with EnergyPlus. Following the recommendations of Agüera et al. in [35], this research used the real weather forecast from an external provider instead of synthetic data. Agüera et al. also found that many papers that employ weather forecast for load forecasting employed test periods of one day or shorter, and they recommended the use of longer testing periods. In the present study, the methodology was validated using a whole month. Regarding the load forecasts, Agüera et al. [35] recommended the introduction of at least outdoor temperature forecasts, and they considered humidity estimations to be valuable. Studies that have accounted for weather forecasts have usually incorporated only outdoor temperature [52], outdoor temperature and relative humidity [34,36] or temperature and solar irradiation [32] but not all influential weather parameters. Previous studies from the authors have shown that weather parameters such as wind speed can be very influential on the building’s load [70]. For this reason, this research introduced forecast data for outdoor temperature, relative humidity, direct normal irradiation, diffuse horizontal irradiation, wind speed and wind direction.

Regarding the results from the case study, first, the probabilistic load forecast was generated based on all 13 months of data. The hourly map of the uncertainty of the heating load for this building and weather provider is presented, and an example of its application is shown. This map shows the probability of having *x* error in the load forecast for each forecast hour (from 1 to 144 h) for different prediction intervals from 10% to 90%. In this case, the map shows clearly that the forecast weather data of the weather provider and location of the study generate an overestimation of the heating load.

The validation of the methodology was performed for the one-day-ahead weather forecast, which is the most common time horizon used, using the PICP and MPIW indicators. The evaluation was performed using April 2020 as the testing period, and the previous months were used to generate the prediction intervals. The results reveal a good performance: the PICP is 83.1%, which was greater than the confidence level (PINC = 80%), with an MPIW of 17.5 kWh. In order to evaluate the influence in the results of the size of the sample used for the creation of the uncertainty map, an analysis was performed gradually increasing the number of months employed and maintaining April 2020 as the testing period. It showed robust and similar results despite the amount of data employed in the uncertainty map creation. In all the cases, PICP values were above the confidence level ranging from 82.2 to 91.5%.

## 6. Conclusions

The present study shows a probabilistic load forecasting methodology based on the use of white-box models developed in EnergyPlus instead of data-driven or black-box models, which are the commonly used prediction models employed in the literature for PLF approaches. White-box models allow to link the results to the physical and architectural building parameters, evaluate the thermal indoor conditions in different time and spatial scales and are more flexible and robust to changes in the building or operation schedules. The proposed methodology allows to build an uncertainty map of the building load prediction, which considers the uncertainty due to the use of weather forecast data, and then apply it to the point load forecast provided by the building simulation model.

The proposed methodology was applied in a case study showing, through the uncertainty map, an easy way to represent the expected hourly probabilistic load forecast error, which is very useful for interpretation by the building’s manager, aggregators, microgrids operators, etc. The results of the evaluation of this methodology showed that for the testing period (April 2020), more than 80% of the actual hourly heating loads values were within the prediction intervals. The influence of the amount of data used for the creation of the uncertainty map was analyzed gradually increasing the number of months employed. It showed that the methodology is robust since similar results, always with PICP values above the confidence level, were obtained despite the amount of data employed. Therefore, the proposed methodology and the resulting map of uncertainty shown in this research are useful tools for applications in which the future load forecast is required, since future predictions always imply weather forecast uncertainties. For example, when predictive control strategies are implemented in a microgrid and the consumer has to provide a day-ahead demand plan with an hourly resolution, which is supposed to be granted within a given confidence interval, the probabilistic load forecast of the building allows the network operators to better make decisions and plan for future smart grids; or when the building participates in a demand response (DR) program, the aggregator that manages the DR events can use a probabilistic load forecast that accounts for uncertainties such as the weather forecast to make an informed decision about which buildings to rely on for the event.

A limitation of the study was that only heating load forecast, and therefore only cold months, were considered since no cooling system was available in the building. In future research, this methodology will be applied and tested in other buildings with both heating and cooling systems in order to assess how the seasonality of the weather data influences the results when the whole year is employed. Furthermore, we are currently analyzing whether the proposed map of uncertainty can be generated for a cluster of buildings with similar architectural characteristics, use patterns and weather conditions. This could be a simple and useful tool for network managers or aggregators in the DR context.

## Figures and Tables

**Table 1:** Error metrics for the point load forecast. Comparison between forecast and real heating load.

Index	Forecast Day 1	Forecast Day 2	Forecast Day 3	Forecast Day 4	Forecast Day 5	Forecast Day 6
MAE (kWh)	9.91	10.50	11.04	11.57	12.43	13.74
MAPE (%)	38.99	41.26	43.56	47.39	51.10	55.84
$R^{2}$ (%)	74.48	70.82	65.99	59.65	49.72	45.66

**Table 2:** Prediction interval coverage probability (PICP) and mean prediction interval width (MPIW) results when using a gradually increasing amount of data for the creation of the uncertainty map. April 2020 is maintained as the testing period.

Months Uncertainty Map	March 2020	February 2020 March 2020	January 2020 March 2020	December 2019 March 2020	November 2019 March 2020	October 2019 March 2020	May 2019 March 2020	April 2019 March 2020	March 2019 March 2020	February 2019 March 2020	January 2019 March 2020	December 2018 March 2020
**N° of months**	1	2	3	4	5	6	7	8	9	10	11	12
**PICP (%)**	91.5	89.9	84.4	82.2	83.1	84.2	83.8	84.3	84.4	84.2	84.0	83.1
**MPIW (kWh)**	27.1	23.1	18.8	18.4	18.3	17.8	17.5	17.2	17.7	17.4	17.7	17.5

## Abbreviations

The following abbreviations are used in this manuscript:

ANN	Artificial neural network
ARIMA	Autoregressive integrated moving average
ARMA	Autoregressive moving average
BEM	Building energy model
BMS	Building management system
CDF	Cumulative distribution function
DR	Demand response
DSM	Demand-side management
EPW	EnergyPlus weather file
HVAC	Heating, ventilation, and air conditioning
Iot	Internet of things
KDE	Kernel density estimation
kWh	Kilowatt hour
LR	Linear regression
MAE	Mean absolute error
MAPE	Mean absolute percentage error
MCM	Monte Carlo method
ML	Machine learning
MPC	Model predictive control
MPIW	Mean prediction interval width
PDF	Probability density function
PICP	Prediction interval coverage probability
PINC	Prediction interval nominal confidence
PLF	Probabilistic load forecast
PI	Prediction intervals
SVM	Support vector machine
$R^{2}$	Coefficient of determination
